# COVID-19 infection in first trimester of pregnancy marked by a liver cytolysis in a woman previously treated by hydroxychloroquine for repeated implantation failure: a case report

**DOI:** 10.1186/s12879-020-05551-0

**Published:** 2020-11-16

**Authors:** F. Lamazou, P. Oger, R. Dieli-crimi, A. Guerin, V. Letouzey, S. Octernaud, V. Place, P. Calès, P. Descamps, L. Delaroche

**Affiliations:** 1 Ramsay Générale de Santé, Hôpital Privé de Parly 2, Institut Fertilité Maternité Parly 2, 21 rue Moxouris, 78150 Le Chesnay, France; 2 Immunology Division, Hospital Universitari Vall d’Hebron (HUVH), Vall d’Hebron Research Institute (VHIR), Department of Cell Biology, Physiology and Immunology, Autonomous University of Barcelona (UAB), Barcelona, Catalonia Spain; 3 Jeffrey Model Foundation Excellence Center, Barcelona, Spain; 4 IVI, Ronda General Mitre, 14, 08017 Barcelona, Spain; 5 Department of Obstetrics and Gynecology, University Hospital Caremeau, Nîmes, France; 6 Medical Radiology center Juras, Paris, France; 7 Hepato-Gastroenterology Department, University Hospital, Angers, France; 8 HIFIH laboratory, UPRES 3859, UNIV Angers, Angers, France; 9 Department of Ob-Gyn, Angers University Hospital, 4 rue Larrey, 49933 Angers Cedex 09, France; 10 Institut des Biomolécules Max Mousseron (IBMM), UMR 5247, CNRS, Université Montpellier, ENSCM, 34093 Montpellier, France; 11 Centre de biologie médicale de l’Hôpital Privé de Parly 2, 21 rue Moxouris, 78150 Le Chesnay, France

**Keywords:** COVID-19, First trimester, Hepatic cytolysis, Liver, Case report, Hydroxychloroquine

## Abstract

**Background:**

In December 2019, a new disease (COVID-19) caused by a novel coronavirus called SARS-CoV-2 emerged in China and spread to many other countries. There is only limited data about the clinical features of COVID-19 during pregnancy, especially in first trimester.

**Case presentation:**

We report a COVID-19 infection in a 35 years-old patient in first trimester of pregnancy and its consequent medical care. At 7 weeks of pregnancy, the patient, who did not have any pregestational comorbidities, complained of intense nausea and asthenia. An important liver cytolysis was discovered with biological perturbations of transaminases levels. No respiratory symptoms were recorded. Classical viral aetiologies and drug-related toxicity were discarded. Because of the aggravation of the symptoms and the occurrence of the breathlessness, the patient was tested for the COVID-19 in a nasopharyngeal swab. The RTq-PCR assay indicated the presence of SARS-CoV-2 RNA. In the absence of severe symptoms, the patient was monitored at home according to the French government guidelines. After a few days, the symptoms resolved without any complications. The pregnancy is still ongoing without any visible sequelae on the foetus so far.

**Conclusions:**

This first case illustrated the difficulty of COVID-19 diagnosis in patients with isolated digestive symptoms in first trimester of pregnancy that could be confused with gravida hyperemesis. Monitoring of pregnancy after an episode of COVID-19 should be strengthened with bimonthly foetal growth ultrasounds and doppler assessments because of the risks for intrauterine growth restriction. Comprehensive data on larger numbers of first trimester gravid women with COVID-19 are required to better understanding the overall impact of SARS-CoV-2 on maternal and birth outcomes.

## Background

In December 2019, a newly identified coronavirus named severe acute respiratory syndrome coronavirus 2 (SARS-CoV-2) emerged in Wuhan, Hubei Province, China [1]. The coronavirus disease 2019 (COVID-19) is mainly transmitted by droplets emitted from the respiratory tract or by handling. The most frequent symptoms are fever (98%), cough (76%), myalgia (44%), expectorations (28%) and headache (8%) [2]. However, severe forms have been described with a severe acute respiratory syndrome particularly in older patients and those who have comorbidities such as diabetes or obesity. The mortality rate of SARS-CoV-2 infection has been evaluated to 1·38% (1·23–1·53), with substantially higher ratios in older age groups [3].

The virus enters cells after binding to membrane angiotensin-converting enzyme II (ACE2) receptor [4]. Therefore, organs with large proportion of cells with ACE2 receptors (lung, heart, esophagus, kidney, bladder, and ileummay) act as target and thus are susceptible to COVID-19 infection [5]. Gastrointestinal symptoms such as diarrhea, vomiting, and abdominal pain have been reported in COVID-19 patients [6]. In addition, patients with COVID-19 can have liver injury with raised enzymes found in blood tests during disease progression [7]. The liver injury could be because of direct viral infection of hepatocytes immune-related injury, or drug hepatotoxicity [8, 9]. There is also suggestion that the virus may bind to cholangiocytes through the ACE2 receptor to dysregulate the liver function [8, 9].

Furthermore, there is currently limited data about the clinical features of COVID-19 during pregnancy [10]. Pregnant women may be more susceptible to COVID-19 since pregnant women, in general, are vulnerable to respiratory infection due to their state of special immune tolerance. Reports suggest that pregnancy is associated with increased risk for intensive care units and receipt of mechanical ventilation compared with non-pregnant women but their risk for death is similar [11]. Moreover, severe acute respiratory syndrome during pregnancy is not associated with an increased risk of spontaneous abortion and spontaneous preterm birth [12]. To date, the risk of vertical transmission of the SARS-CoV-2 to the foetus is not well known even if few reports seems to be reassuring [12–15]. Some new-borns, whose mothers were infected by the SARS-CoV-2 during late pregnancy, seemed in good health [16, 17]. However, some perinatal COVID-19 infection with adverse effects on newborns such as fetal distress, premature labor, respiratory distress, thrombocytopenia accompanied by abnormal liver function have been reported [15, 18].

We report a first case of COVID-19 infection in a 7 weeks pregnant woman suffering from isolated liver damage with cytolysis and the subsequent medical care. This study was approved by the French Ethics Committee (IRB 00010835). A copy of the written consent is available for review by the Editor-in-Chief of this journal.

## Case presentation

A 35-year-old Caucasian woman developed a COVID-19 infection during the 7th week of pregnancy.

The pregnancy was achieved after a frozen-thawed embryo transfer performed on the 20 February 2020 in a gametes donation programme after autologous repeated implantation failure (RIF). The patient was monitored according to a substituted protocol with daily 6 mg of oral oestradiol (Provames®, SERB, France), 800 mg of intravaginal progesterone (Utrogestan®, Besins, France). Because of the RIF history, an immunological treatment with 7·5 mg of prednisone (Cortancyl®, Sanofi Aventis, France), 400 mg of hydroxychloroquine (Plaquenil®, Sanofi Aventis, France), 100 mg of aspirin (Aspegic®, Sanofi Aventis, France), 0·4 mL of enoxaparin (Lovenox®, Sanofi Aventis, France) and 0·2 mL of filgrastim (Growth Hormon (GH)), (Neupogen®, Amgen, Holland) per day was started 10 months before the embryo transfer. The patient did not have any pregestational comorbidities such as hypertension, diabetes, or cardiovascular disease.

At 7 weeks of amenorrhea (WA), namely day zero (D0) of the symptoms, the patient started to complain about nausea, asthenia, and symptoms of gastroesophageal reflux. At day seven (D7), liver tests showed that aspartate aminotransferase (AST), alanine aminotransferase (ALT) and gamma-glutamyl transpeptidase (GGT) were 3, 5 and 2 times the upper level of normal (ULN) respectively (Table 1). At day ten (D10), symptoms worsened (asthenia, permanent nausea, mild and diffuse abdominal pain, shortness of breath when exercising), but the patient did not complain about fever (37·5 °C). The clinical exam was normal, and an ultrasound scan at 8WA + 2 days confirmed an evolutive intra uterine pregnancy without any sign of molar pregnancy. The length of the embryo was 20·9 mm corresponding to the gestational age with a perceptible heartbeat. Treatment was changed: oral oestradiol was switched to oestrogenic patches of 100 mg per 2 days (Vivelledot®, Sandoz, France), and prednisone was increased to 10 mg per day. Hydroxychloroquine was stopped. Serology for viral hepatitis A, B, C, E, cytomegalovirus (CMV), and Epstein Barr virus (EBV) returned negative. The new blood tests showed white blood cell count of 13·0 G/L with neutrophil count of 10·88 G/L and lymphocyte count of 1·39 G/L. Transaminases AST and ALT increased to 4 and 8 ULN, respectively and, GGT to 4 ULN but alkaline phosphatases (PAL) and lipase were still normal. Electrolytes and creatinine were also normal. D-dimers were slightly increased at 0·506 μg/mL. C-reactive protein (CRP) was 14 mg/L. Biological results are resumed in Table 1. Liver ultrasound scan found sludge in the gallbladder which had a thin wall whereas the bile ducts and liver were normal.

**Table 1:** Course of blood tests between day 7 and day 31 after the first symptoms

	Reference values	Day 7	Day 10	Day 11	Day 13	Day 16	Day 18	Day 22	Day 31
White blood cell count (G/L)	4·0–11·0	6·6	**13**·**0**	**14**·**2**	**17**·**0**	**15**·**4**	10·0	9·3	7·9
Neutrophil count (G/L)	1·50–7·50	5·23	**10**·**88**	**12**·**50**	**12**·**45**	**12**·**74**	**7**·**74**	6·46	5·44
Lymphocyte count (G/L)	1·10–4·40	**1**·**00**	1·39	1·84	2·24	2·11	1·74	2·23	1·75
Platelets count (G/L)	150–400	179	188	211	237	238	265	291	233
CRP (mg/L)	< 5	4	**14**	**18**	**8**	1	1	2	4
Procalcitonine (ng/mL)	≤ 0·12	–	< 0·12	–	–	–	–	–	
AST (IU/L)	5–34	**96**	**137**	**130**	**94**	**41**	**26**	**19**	17
ALT (IU/L)	< 55	**296**	**434**	**404**	**364**	**211**	**99**	**67**	31
GGT (IU/L)	9–36	**87**	**117**	**121**	**109**	**87**	**64**	**53**	32
PAL (IU/L)	40–150	74	90	78	88	76	76	57	45
Total Bilirubin (mg/L)	3·0–10·0	3·4	4·7	4·9	5·7	5·1	5·3	5·4	3·6
Lipase (IU/L)	8–78	55	69	–	–	–	–	–	
Prothrombin index (%)	70–150	–	89	–	93	97	95	93	91
D-dimers (ng/mL)	< 500	–	506	–	–	–	–	–	
Sodium (mmol/L)	136–145	–	137	–	–	–	–	–	
Potassium (mmol/L)	3·5–5·2	–	3·4	–	–	–	–	–	
Creatinine (μmol/L)	40–90	–	85	74	73	65	65	73	65

*ALT* Alanine aminotransferase, *AST* Aspartate aminotransferase, *GGT* Gamma-glutamyl transpeptidase, *PAL* Alkaline phosphatases, *CRP* C-reactive protein

In the COVID-19 epidemic context, it was decided to screen the patient for COVID-19 infection. The RTq-PCR assay indicated the presence of SARS-CoV-2 RNA in the nasopharyngeal swab. In the absence of signs of severity, it was decided to monitor the patient at home according to the French government guidelines. No symptomatic treatment was given to the patient for the COVID-19 infection. However, the luteal phase support treatment was maintained. Gradually, the severity of nausea, asthenia and shortness breath diminished, and the blood tests returned to normality. Ultrasound scans performed on the 11th week of amenorrhea confirmed the satisfactory evolution of the pregnancy: the foetus had followed a normal growth with a size of 51·0 mm and the morphological exam did not reveal any malformations and the nuchal translucency was normal at 1·3 mm.

## Discussion and conclusion

To our knowledge, we report for the first time a COVID-19 infection marked by a cytolysis diagnosed in first trimester of pregnancy (7th to 11th week of amenorrhea) with spontaneous resolution, and no visible sequelae on the foetus so far.

Currently, there are limited data about COVID-19 infections during pregnancy. Some authors reported cases from the 24th week of pregnancy with outcomes generally favourable for both mothers and foetuses. There seems to be no evidence of vertical transmission [12–16], but previous experience with infections caused by similar virus such as SARS and MERS, indicates that intra-uterine transmission is not an exclusive cause of foetal morbidity and mortality [14, 19]. There have been adverse outcomes reported in the infants of women with COVID-19, although causality from the infection cannot yet be determined [14, 18]. These include preterm labor and delivery, premature rupture of membranes, intrauterine growth restriction, low birth weight, intrauterine fetal distress, feeding intolerance, asphyxia, pneumonia and respiratory distress, and stillbirth [14]. Moreover, the available literature on possible placental changes and consequences for the course of pregnancy during an infection in gravidate is very limited, even if recent articles reported no increased risk of spontaneous abortion [12]. Samples from amniotic fluid, umbilical cord blood, breast milk and throat swabs of the new-borns after delivery were reported negative for the SARS-CoV-2 [12, 18]. As recommended by the guidelines for the management of laboratory-confirmed SARS-CoV-2 pregnant women [15], this patient will be monitored with bimonthly foetal growth ultrasounds and doppler associated to a morphological analysis of the foetus. We chose to add to this monitoring a hepatic assessment also bimonthly.

This case illustrated the difficulty of the diagnosis of the COVID-19 infection in a patient who suffered from nausea and asthenia in the first trimester of pregnancy, without any respiratory symptoms. The risk was to miss the COVID-19 diagnosis and consider it was a gravida hyperemesis which is classically observed in the first trimester of the pregnancy. The changes in liver enzymes alerted us because transaminases levels are usually normal in the beginning of the pregnancy. Transaminases perturbations can be observed as adverse side effects of drugs, especially after oestrogen and hydroxychloroquine administration as described in rare cases [20]. In our case, we cannot rule out drug-induced hepatitis. However, hydroxychloroquine was stopped, and oestradiol was reduced without any clinical improvement. Hydroxychloroquine was administrated during 1 year because of the RIF history in order to improve the implantation process as previously described [21]. In the recent emergence of the COVID-19, hydroxychloroquine has been proposed as an optional therapy [22]. It has been described that hydroxychloroquine could reduce the SARS-CoV-2 cell invasion and control of viral replication [23, 24]. It could also inhibit the toll-like receptors activation and control the cytokine storm due to secondary hemophagocytic lympho-histiocytosis observed in severe cases. But hydroxychloroquine efficacy remains controversial. We questioned whether hydroxychloroquine administered during 1 year in our patient could have partially reduced the progression of the SARS-CoV-2 and decreased the risk of severe form [11].

Isolated digestive symptoms have already been reported in COVID-19 patients [25]. As reported by Luo and colleagues, among 1141 patients infected by the SARS-CoV-2, 16% presented digestive symptoms with appetite loss, 66% nausea and vomiting, 37% diarrhoea and 25% abdominal pain without any other symptoms. The review by Bangash et al. [26] on seven studies reported elevated aminotransferases and bilirubin, with a higher prevalence of abnormal aminotransferases levels in severe COVID-19 infections. Although abnormal liver function tests seem common in COVID-19 patients, the impairment of liver function does not seem a prominent feature of COVID-19 and may not have serious clinical consequences [9, 10, 27]. It has been proposed that COVID-19 can cause direct hepatocyte damage, but other authors believe that liver damage is due to virally induced cytotoxic T cells and the induction of dysregulated innate immune response or drug toxicity. The pregnancy bias towards T-helper 2 (Th2) system dominance which protects the foetus, leaves the mother vulnerable to viral infections, which are more effectively contained by the Th1 system [26]. In our case, the patient did not present any lymphopenia and the CRP level was only slightly elevated. Lymphopenia and elevated markers of inflammation have been reported as current biological signs of COVID-19 infection, even in pregnancies [28]. However, our patient was treated by corticoids and GH during this period, that could explain the lack of these abnormalities. In addition, the white blood cell count is often higher in the first trimester of pregnancy. Furthermore, hepatic dysfunction in severe COVID-19 is often accompanied by greater activation of coagulative and fibrinolytic pathways, relatively depressed platelet counts, increased neutrophil counts and neutrophil to lymphocyte ratios, and high ferritin levels [18]. We did not observe coagulative dysfunctions, probably because an antithrombotic therapy (0·4 mL of enoxaparin (Lovenox®, Sanofi Aventis, France)) was administered considering the RIF history. To prevent thrombotic and thromboembolic complications, an antithrombotic therapy is recommended in COVID-19 patients [29, 30].

In conclusion, we report for the first time a case of COVID-19 in the first trimester with spontaneous resolution and no sequalae on the foetus so far. We believe that these findings will contribute to better understanding the overall impact of SARS-CoV-2 on maternal and birth outcomes in order to establish guidelines for the management of laboratory-confirmed SARS-CoV-2 pregnant women.

## Data Availability

All the data’s (biology, ultrasound scan) are available.

## Abbreviations

ALT: Alanine aminotransferase
AST: Aspartate aminotransferase
CMV: Cytomegalovirus
CRP: C-reactive protein
D: Day
EBV: Epstein Barr virus
GGT: Gamma-glutamyl transpeptidase
GH: Growth hormone
MERS: Middle East Respiratory Syndrome
PCR: Polymerase chain reaction
RIF: Repeated implantation failure
SARS: Severe Acute Respiratory Syndrome
ULN: Upper level of normal
WA: Weeks of amenorrhea
